# Effect of Albendazole on Human Hydatid Cysts: An Ultrastructural Study

**DOI:** 10.1155/1990/47243

**Published:** 1990

**Authors:** K. Sylvia Richards, D. L. Morris

**Affiliations:** 1 Department of Biological Sciences University of Keele Staffs. ST5 5BG United Kingdom; 2 Department of Surgery University Hospital Nottingham NG7 2UH United Kingdom

## Abstract

Five patients with hepatic (3), pelvic (1) or spinal (1) hydatid cysts received 10 mg/kg/d albendazole for 1–3 months prior to surgery. Daughter cysts were present in the spinal hydatid and in one patient with hepatic disease. Electron microscope examination of the cyst tissue of the pelvic and the 2 hepatic cysts lacking daughter cysts showed no evidence of germinal layer, and the protoscoleces were dead. The primary cyst of the hepatic hydatid with daughter cysts (1 month therapy) was also judged dead but some pieces of the daughter cyst germinal layer appeared normal and had unaffected protoscoleces. The daughter cyst tissue of the spinal hydatid (3 month therapy) appeared normal and the protoscoleces viable. In view of the undetermined viability of human hydatids before chemotherapy, treatment of longer than 1 month is advocated for hepatic cysts, particularly if daughter cysts are present, and longer therapy is indicated for spinal disease.


*HPB Surgery* 1990, Vol. 2 pp. 105–113
Reprints available directly from the publisher
Photocopying permitted by license only

© 1990 Harwood Academic Publishers GmbH
Printed in the United Kingdom

### EFFECT OF ALBENDAZOLE ON HUMAN HYDATID CYSTS: AN ULTRASTRUCTURAL STUDY

K. SYLVIA RICHARDS* and D.L. MORRIS**

**Department of Biological Sciences, University of Keele, Staffs. ST5 5BG, UK*
***Department of Surgery, University Hospital, Nottingham NG7 2UH, UK*

*(Received 20 July 1988)*

Five patients with hepatic (3), pelvic (1) or spinal (1) hydatid cysts received 10 mg/kg/d albendazole for 1–3 months prior to surgery. Daughter cysts were present in the spinal hydatid and in one patient with hepatic disease. Electron microscope examination of the cyst tissue of the pelvic and the 2 hepatic cysts lacking daughter cysts showed no evidence of germinal layer, and the protoscoleces were dead. The primary cyst of the hepatic hydatid with daughter cysts (1 month therapy) was also judged dead but some pieces of the daughter cyst germinal layer appeared normal and had unaffected protoscoleces. The daughter cyst tissue of the spinal hydatid (3 month therapy) appeared normal and the protoscoleces viable. In view of the undetermined viability of human hydatids before chemotherapy, treatment of longer than 1 month is advocated for hepatic cysts, particularly if daughter cysts are present, and longer therapy is indicated for spinal disease.

### INTRODUCTION

Although surgery remains the primary treatment for human hydatid disease, there is a growing interest in medical treatment, particularly where resection is contraindicated due to disseminated disease, the condition of the patient or in an adjuvant setting.

Mebendazole was the first drug shown to have an effect[1] albeit inconsistent[2–4] and more encouraging results have been achieved using albendazole[5–7].

Efficacy is generally judged clinically or monitored radiographically to determine cyst regression. When chemotherapy has been followed by surgery, the viability of the protoscoleces within the cyst has been assessed, but less often has the germinal layer of the hydatid been examined histologically, and reports of the ultrastructure of this layer after therapy have been more rare. Verheyen[8] compared human untreated and mebendazole-treated hydatid germinal layer using electron microscopy, and Al-Dabagh *et al.*[9] and Gemmell *et al.*[10] reported the effect of mebendazole on the ultrastructure of sheep hydatids. Morris *et al.*[11] included an ultrastructural study in their report on albendazole treatment of pulmonary hydatid cysts in naturally infected sheep. More recently, Rahemtulla *et al.*[12] used histology and electron microscopy to examine a resected human hydatid after albendazole therapy, but no ultrastructural details of the germinal layer were given.

The present communication reports the results of an ultrastructural examination of the germinal layer of cysts (primary and daughter where present) removed from 5 patients, all of whom had received albendazole therapy prior to surgery.

### MATERIALS AND METHODS

*Chemotherapy prior to surgery.* The five patients (#1–5) all received albendazole therapy prior to surgery, and the details of cyst site, dosage, duration of therapy and viability of protoscoleces at surgery (assessment:eosin exclusion/passage into gerbils) are given in Table 1.

*Electron Microscopy.* Pieces of germinal layer and attached laminated layer were carefully taken from hydatid cysts removed at surgery from the 5 patients. Daughter cyst material, where present, was processed separately from primary cyst tissue.

Primary fixation was in 3% glutaraldehyde in 0.1M phosphate buffer for a minimum of 6 h, followed by at least 2 buffer washes prior to post-fixation in buffered 1% osmium tetroxide for 1 h. Dehydration was in a graded acetone series and embedment was in Spurr's resin.

A minimum of 6 pieces/cyst were examined as follows: routine 0.5 $\mu$m sections of each tissue piece were stained with Toludine blue and examined by light microscopy. Ultrasections, from chosen areas of these blocks, were cut and stained with aqueous uranyl acetate followed by lead citrate and examined in a Philips 200.

**Table 1** Cyst site, therapy and protosolex viability

| *Patient* | *Cyst site and type* | *Dose (divided) mg/kg/d* | *Length of therapy (Months)* | *Protosolex viability at resection* |
|---|---|---|---|---|
| 1 | Liver P | 10 | 1 | Dead |
| 2 | Pelvis P | 10 | 3 | Dead |
| 3 | Liver P | 10 | 2 | Dead |
| 3 | Spine* D | 10 | 3 | Alive |
| 4 | Liver P,D | 10 | 1 | Alive |

P, Primary cyst; D, Daughter cyst; *, Recurrence

### RESULTS

In 2 of the 5 patients (#4,5), live disease, as determined by viable protoscoleces, was present (Table 1). Both of these patients had daughter cysts.

The ultrastructure of the germinal layer of untreated human hydatid has been described[8,13] and is similar to that reported from other intermediate hosts[14,15]. It is typically cestode in organisation with a distal cytoplasm connected by internuncial processes to nucleated tegumentary cytons that house the mitochondria, Golgi stacks, vesicles etc. Extending from the tegumentary and myocytons are large, irregular, glycogen-containing processes, and arising from the distal cytoplasm and projecting into the laminated layer are truncated microtriches (i.e. without spines but possessing a sub-apical circular baseplate[16]).

Pieces of germinal layer from primary cysts, and daughter cysts where present, from the 5 patients (#1–5) were examined at the light (LM) and electron (EM) microscope level. A minimum of 6 pieces/cyst were examined.

*Patient* #1.**Primary** hepatic cyst: At neither LM nor EM level was there evidence of germinal layer tissue.

*Patient* #2. **Primary** pelvic cyst: At neither LM nor EM level was there evidence of germinal layer tissue.

*Patient* #3. **Primary** hepatic cyst: At neither LM nor EM level was there evidence of germinal layer tissue.

*Patient* #4. **Daughter** spinal cysts: At the LM level, intact germinal layer was present on 5 of the 6 pieces examined; on the 6th there was, adjacent to the laminated layer, an ill-defined zone that failed to stain adequately with Toluidine blue.

At the EM level, Figures 1 and 2 are typical of the intact germinal layer, and glycogen reserves and comparatively large mitochrondria with dense matricies can be seen in Figure 1, whereas the more impoverished germinal layer seen in Figure 2 possesses large mitochondria, laminated residual bodies and sparse glycogen deposits.

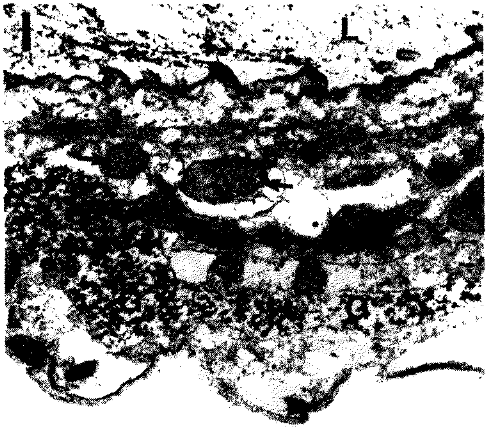

**Figure 1** Spinal Daughter Cyst (Patient #4). Germinal layer showing mitochondria(arrows) and glycogen reserves(G). L,laminated layer. × 11900.

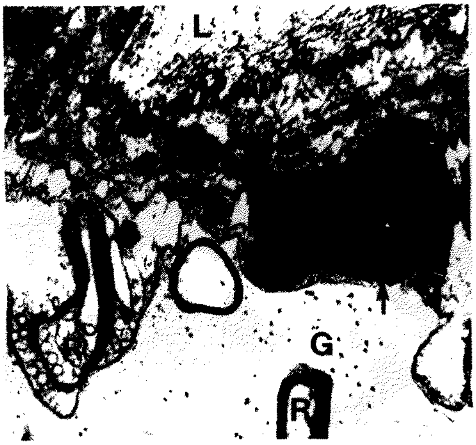

**Figure 2** As Figure 1 but a more impoverished germinal layer with very large mitochondria(arrows), sparse glycogen(G) and lamellated residual bodies(R). L,laminated layer. × 11900.

The ill-defined germinal layer of the 6th tissue piece completely lacked integrity at the EM level. Occasional vesicles were seen in contact with the laminated later (as in Figure 4), and lying internal to this, but detached from it, were highly vesiculated fragments of tissue as shown in Figure 3.

*Patient* #5. **Primary** and **Daughter** hepatic cysts:

**Primary** cyst. At the LM level, 3 tissue pieces showed no evidence of germinal layer; the other 3 possessed an ill-defined zone that stained inadequately.

Where there was no evidence of germinal layer tissue at LM, the EM study revealed either no tissue remnants in contact with the laminated layer or, more frequently, a narrow zone of vesicles (Figure 4). Occasionally, small regions of necrotic tissue, in which myelin figures, lipid droplets and small autophagacytosed glycogen deposits could be seen, remained in contact with the laminated layer (Figure 5). More rarely, profiles as in Figure 6 were observed.

**Daughter** cyst tissue. At the LM level, no germinal layer was evident in 4 of the 6 pieces examined, the remaining 2 pieces possessing an intact germinal layer with attached brood capsules in which protoscoleces were present.

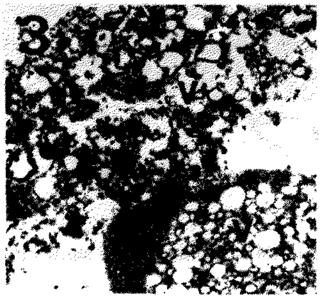

**Figure 3** As Figure 1 but no intact germinal layer present. Internal to the laminated layer are highly vesiculated tissue fragments(V). × 7170.

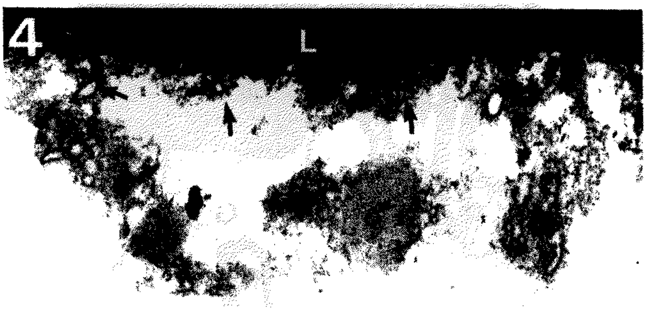

**Figure 4** Hepatic Primary Cyst (Patient #5). A narrow zone of vesicles(arrows) remains in contact with the laminated layer(L). × 15200.

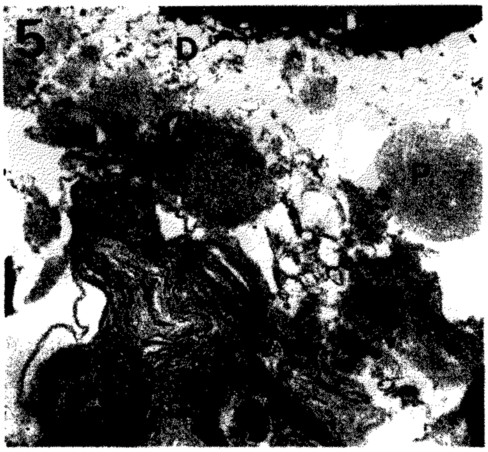

**Figure 5** As Figure 4. Necrotic tissue remains in contact with the laminated layer(L). D, distal cytoplasm; P, lipid droplet; Y, myelin figures; arrow, autophagocytosed glycogen deposits. × 9250.

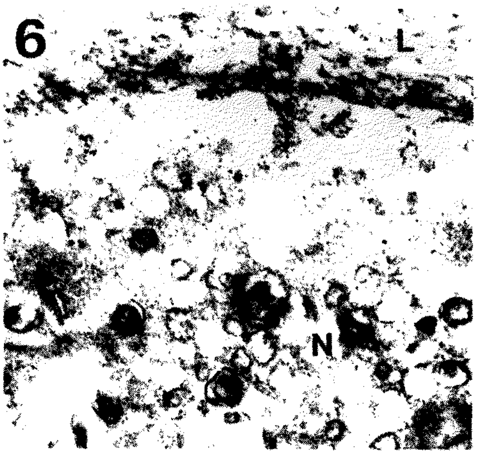

**Figure 6** As Figure 4. Necrotic tissue(N) in contact with the laminated layer(L). × 28200.

EM examination of the 4 pieces lacking germinal layer at the LM level revealed no tissue remnants whereas the other 2 pieces produced profiles similar to that shown in Figure 7 in which a nucleus, glycogen deposits, lipid deposits, mitochrondria and mucopolysaccharide residual bodies can clearly be seen. Occasional cell masses were observed (not illustrated) and were interpreted as incipient brood capsules. The ultrasections passed through the wall of developed brood capsules, the tissue of which appeared normal and similar to that recorded by Rogan and Richards[17] from control murine and equine tissue. Profiles of protoscoleces were observed and Figure 8 shows part of a protoscolex soma. The tissue is indistinguishable from control material taken from other intermediate hosts[16–18].

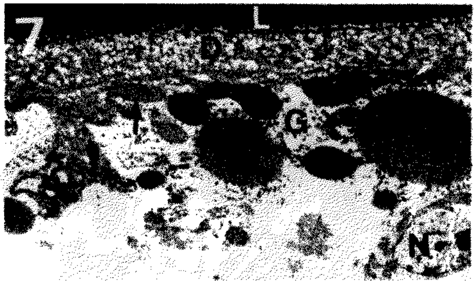

**Figure 7** Hepatic Daughter Cyst (Patient #5). An intact germinal layer is present. L, laminated layer; D, distal cytoplasm; N, nucleus; G, glycogen; P, lipid; arrow, mitochondrion; S, mucopolysaccharide residual body. × 3570.

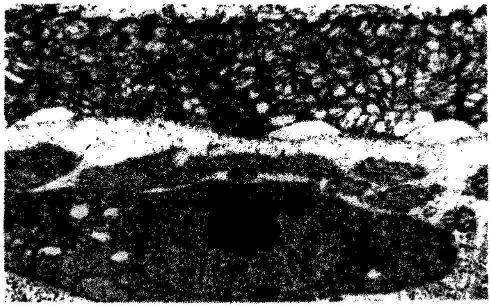

**Figure 8** Outer part of the soma of a protoscolex within a hepatic daughter cyst (Patient#5). D, distal cytoplasm; T, tegumentary cyton; N, nucleus; G, glycogen. × 9250.

### DISCUSSION

Although the primary cyst wall present in 4 of the 5 patients showed no evidence of intact germinal layer tissue, it would be uncautious to attribute this, unequivocally, to albendazole therapy.

In cases of human hydatid, reliable methods of viability assessment prior to therapy are not available. Scanning methods often fail to reveal dead cysts[12] and the germinal layer does not necessarily collapse. These problems were acknowledged by Rahemtulla *et al.*[12] but they nevertheless attributed the dead cyst at resection to albendazole therapy.

In field trials using sheep[11,19] aspiration of pulmonary cysts before therapy is possible, but this only reveals the viability or otherwise of the protoscoleces and does not indicate the status of the germinal layer.

The vesicular remnants persisting in contact with the laminated layers of the primary cysts are similar to those currently being reported in a study on the long-term effect of albendazole on sheep hydatids[19] and recorded in secondary murine cysts treated *in vivo* with praziquantel[20].

In both sheep and humans, the age of the infection is unknown, and disintegration of the germinal layer through natural death cannot be excluded; in young infections in mouse the collapse and fragmentation of the layer is certainly more likely to have been drug-related.

Daughter cysts were present in patients #4 and 5 and intact germinal layer was observed in both, together with, in patient #5, structurally undamaged protoscoleces. It would appear that therapy of 1 month in patient #5 was insufficient to allow adequate penetration and maintenance of effective drug levels within the daughter cysts to achieve destruction of the germinal layer and protoscoleces. Patient #4 had, however, received albendazole for 3 months prior to surgery. In this case, the apparent inefficacy of the drug might be attributed to the possibly privileged site in which this hydatid occurred. Whilst albendazole does enter the CSF in both man and sheep, the concentrations are considerably less than those present in the serum.

The evidence gained from this study would suggest that albendazole therapy of 1 month is probably not sufficient to kill germinal layer reliably, especially that of daughter cysts, although patients #1–3 (with only primary cysts) had no viable tissue at the end of such a treatment time. In view of the uncertainty of the viability status of the germinal layer and protoscoleces prior to treatment, the duration of therapy should be longer than 1 month. In cases of hydatid in the central nervous system, much longer periods of therapy would seem necessary.

### *Acknowledgements*

The financial support of Smith Kline & French is gratefully acknowledged.

### *References*

1. Bekhti, A., Nizet, M., Capron, M., *et al.* Chemotherapy of human hydatid disease with mebendazole: follow up of 16 cases, *Acta Gastro-ent. Belg.*, 1980; **43**, 48–65.
2. Braithwaite, P.A., Long-term high-dose mebendazole for cystic hydatid disease of liver: failure in two cases, *Aust.N.Z.J.Surg.*, 1981; **51**, 23–27.
3. Schantz, P.M., Van den Bossche, H. & Eckert, J., Chemotherapy for larval *Echinococcus* in animals and humans: report of a workshop, *Z.Parasitenkd*, 1982; **67**, 5–26.
4. Davis, A., Pawlowski, Z.S. and Dixon, H., Multicentre clinical trials of benzimidazole carbamates in human echinococcosis, *Bull. WHO*, 1986; **64**, 383–388.
5. Saimot, A.G., Meulemans, A., Cremieux, A.C., *et al.*, Albendazole as a potential treatment for human hydatidosis, *Lancet*, 1983; **2**, 652–656.
.6 Morris, D.L., Dykes, P.W., Dickson, B., *et al.*, Albendazole in hydatid disease, *Brit. Med. J.* 1983; **286**, 103–104.
7. Morris, D.L., Dykes, P.W., Marriner, S.E., *et al.*, Albendazole—objective evidence of response in human hydatid disease, *J. Amer. Med. Ass.*, 1985; **253**, 2053–2057.
8. Verheyen, A., *Echinococcus granulosus:* the influence of mebendazole therapy on the ultrastructural morphology of the germinal layer of hydatid cysts in humans and mice, *Z. Parasitenkd*, 1982; **67**, 55–65.
9. Al-Dabagh, M.A., Al-Moslih, M.I., Verheyen, A., *et al.*, The effect of mebendazole on sheep hydatids as demonstrated by electron microscopy, *J. Parasitol.*, 1981; **67**, 709–712.
10. Gemmell, M.A., Parmeter, G.N., Sutton, R.J. and Khan, N., Effect of mebendazole against *Echinococcus granulosus* and *Taenia hydatigena* cysts in naturally infected sheep and relevance to larval tapeworm infections in man, *Z. Parasitenkd.*, 1981; **64**, 135–147.
11. Morris, D.L., Clarkson, M.J., Stallbaumer, M.F., *et al.*, Albendazole treatment of pulmonary hydatid cysts in naturally infected sheep: a study with relevance to the treatment of hydatid cysts in man, *Thorax*, 1985; **40**, 453–458.
12. Rahemtulla, A., Bryceson, A.D.M., McManus, D.P. and Ellis, D.S., Albendazole in the treatment of hydatid disease, *J. Roy. Soc. Med.*, 1987; **80**, 119–120.

13. Bortoletti, G. and Ferretti, G., Investigation on larval forms of *Echinococcus granulosus* with electron microscope, *Riv. Parassit.*, 1973; **34**, 89–110.
14. Bortoletti, G. and Ferretti, G., Ultrastructural aspects of fertile and sterile cysts of *Echinococcus granulosus* developed in different species, *Int. J. Parasitol.*, 1978; **8**, 421–431.
15. Richards, K.S., Arme, C. and Bridges, J.F., *Echinococcus granulosus equinus:* variation in the germinal layer of murine hydatids and evidence of autophagy, *Parasitology*, 1984; **89**, 35–47.
16. Rogan, M.T. and Richards, K.S., *in vitro* development of hydatid cysts from posterior bladders and ruptured brood capsules of equine *Echinococcus granulosus, Parasitology*, 1986; **92**, 379–390.
17. Rogan, M.T. and Richards, K.S., *Echinococcus granulosus:* changes in the surface ultrastructure during protoscolex formation, *Parasitology*, 1987; **94**, 359–367.
18. Morris, D.L., Taylor, D., Daniels, D. and Richards, K.S., Determination of minimum effective concentration of praziquantel in *in vitro* cultures of protoscoleces of *Echinococcus granulosus, Trans. Roy. Soc. Trop. Med. Hyg.*, 1987; **81**, 494–497.
19. Morris, D.L., Richards, K.S., Clarkson, M.J. and Taylor, D.H., Long term results of albendazole therapy of *Echinococcus granulosus*—clinical and experimental results, *J. Amer. Med. Ass.* (submitted).
20. Richards, K.S., Morris, D.L., Daniels, D. and Riley, E.M., *Echinococcus granulosus:* the effects of praziquantel, *in vivo* and *in vitro*, on the ultrastructure of equine strain murine cysts, *Parasitology*, 1988; **96**, 323–336.

*(Accepted by S. Bengmark 18 September 1988)*

### INVITED COMMENTARY

Whilst surgery is still the primary method of treatment for hydatid disease, chemotherapy is increasingly recognised as an important adjunct or, in some instances, alternative. The benzimidazole carbamates mebendazole and albendazole has now been used extensively in this role, with the latter drug showing considerably more promise. This probably relates to the much higher plasma levels (about 100 times higher) of the metabolite of albendazole achieved following comparable oral doses of the two compounds.

In the majority of trials of the effectiveness of albendazole against human hydatid disease 10 mg/Kg/day has been the dosage used but the duration and spacing of courses of treatment has not been standardised. The efficacy of albendazole has usually been assessed by evidence of cyst regression or by determinations of protoscolex viability following surgical removal of the cyst. It is highly desirable that studies which will provide information on the mode of action of such compounds on hydatid cysts, especially on the germinal layer, are performed if a rational basis for dosages and duration of therapy is to be developed. The approach used in the study reported in this manuscript by Richards and Morris provides some observations of this kind but, as the authors state, it is not possible to unequivocally attribute the effects observed to albendazole therapy, especially when such small numbers of patients are involved. Whether the ultrastructural changes found in the non viable cysts are due to albendazole or not, the paper does provide useful descriptions of apparently normal and abnormal hydatid cysts from humans which will be valuable for comparative purposes. In this respect it might have been advantageous to include photomicrographs from the material removed from the other three patients as well or to state that the ultrastructure of those cysts was identical to that of the apparently non-viable material from patients 4 and 5.

From the results of this study it does seem clear that one month of treatment at 10mg/Kg/day is probably insufficient and this would appear to be supported by clinical trials in which cyst regression was used as a criterion of success.[1] Even the three month course received by patient No. 4 was inadequate and, though it may be true that the cyst was in a "privaleged site" less accessible to the active drug, it needs to be borne in mind that four months therapy has not always been successful, even in liver.[1] It is for this reason of varied response that more studies are needed, both to determine the effects of albendazole and other chemotherapeutic agents on cysts in different sites and to determine if different hydatid strains respond differently. This paper provides a useful basis for comparison with future investigation.

### *Reference*

1. Todorov, T. *et al.*, 1988, *Trans. Roy. Soc. Trop. Med. Hyg.* **82**, 453–459.

J.C. Walker
Medical Parasitology Unit
Department of Infectious Diseases and Microbiology
Westmead Hospital, Westmead 2145, Australia

